# Testing encoding specificity and the diagnostic feature-detection theory of eyewitness identification, with implications for showups, lineups, and partially disguised perpetrators

**DOI:** 10.1186/s41235-021-00276-3

**Published:** 2021-03-03

**Authors:** Curt A. Carlson, Jacob A. Hemby, Alex R. Wooten, Alyssa R. Jones, Robert F. Lockamyeir, Maria A. Carlson, Jennifer L. Dias, Jane E. Whittington

**Affiliations:** 1 Texas A&M University –Commerce, PO Box 3011, Commerce, TX 75429 USA; 2 Hollins University, Roanoke, USA; 3 Tarleton State University, Stephenville, USA; 4 Holy Family University, Philadelphia, USA

**Keywords:** Eyewitness identification, Diagnostic feature-detection theory, Encoding specificity, Simultaneous lineup, Showup

## Abstract

The diagnostic feature-detection theory (DFT) of eyewitness identification is based on facial information that is diagnostic versus non-diagnostic of suspect guilt. It primarily has been tested by discounting non-diagnostic information at retrieval, typically by surrounding a single suspect showup with good fillers to create a lineup. We tested additional DFT predictions by manipulating the presence of facial information (i.e., the exterior region of the face) at both encoding and retrieval with a large between-subjects factorial design (*N* = 19,414). In support of DFT and in replication of the literature, lineups yielded higher discriminability than showups. In support of encoding specificity, conditions that matched information between encoding and retrieval were generally superior to mismatch conditions. More importantly, we supported several DFT and encoding specificity predictions not previously tested, including that (a) adding non-diagnostic information will reduce discriminability for showups more so than lineups, and (b) removing diagnostic information will lower discriminability for both showups and lineups. These results have implications for police deciding whether to conduct a showup or a lineup, and when dealing with partially disguised perpetrators (e.g., wearing a hoodie).

## Significance

DNA exoneration cases have revealed the prevalence of mistaken eyewitness identifications, and it is critical to develop theory-driven approaches to improving eyewitness identification accuracy. According to diagnostic feature-detection theory (DFT), eyewitnesses assess suspect guilt by evaluating facial information that matches their memory for the perpetrator but is not also shared by innocent lineup members. We tested several DFT predictions by manipulating the presence of facial information at both encoding (analogous to a perpetrator wearing a hoodie) and retrieval (analogous to police deciding whether to have everyone in a lineup wear a hoodie). By adding this encoding manipulation, we also tested a popular cognitive theory known as encoding specificity, which predicts that eyewitness performance should be superior when encoding conditions match retrieval conditions (e.g., perpetrator wore a hoodie and everyone in lineup has a hoodie). A nationwide sample of participants viewed either a full face or the internal region only and were later tested with a showup or lineup containing full faces or only internal regions. We supported DFT by replicating the lineup advantage over showups, and we supported encoding specificity such that match conditions were generally superior to mismatch conditions. We also confirmed DFT predictions that (a) removing diagnostic information will harm performance and (b) adding non-diagnostic information will harm showups more than lineups. These results provide additional support for DFT as a powerful theory of eyewitness decision-making.

## Background

Mistaken eyewitness identification (ID) is a factor in approximately 71% of the convictions revealed by DNA exoneration in the USA (Innocence Project 2020). This problem has resulted in a great deal of research over the last few decades (e.g., Wells 1978; see reviews by Gronlund and Carlson 2013, and Wells et al. 2006), and the study of eyewitness ID extends back much further (Arnold 1906; Münsterberg 1908). However, from the beginning there was a general lack of theoretical guidance (Bornstein and Penrod 2008; Gronlund and Benjamin 2018). This has resulted in calls for more eyewitness ID research undergirded by cognitive theory generally (e.g., Dianiska et al. 2020; Lane and Meissner 2008), and signal detection theory specifically (SDT; Green and Swets 1966; Wixted and Mickes 2012). Our goal is to test a quantitative theory based in SDT known as diagnostic feature-detection theory (DFT; Wixted and Mickes 2014). It makes predictions about eyewitness discriminability,^1^ and there is an important distinction between theoretical and empirical discriminability (see Wixted and Mickes 2018, for a review). Before we can adequately describe the theory, how it has been tested in the literature, and our own novel testing methods, we will briefly explain these concepts.

### Discriminability

Theoretical discriminability involves the underlying and unobservable memory signals in the mind of an eyewitness and it is often represented by a statistical measure known as *d'* (Green and Swets 1966; Macmillan and Creelman 2005). This measure is derived by calculating the distance between the means of two Gaussian distributions: target stimuli (i.e., guilty suspects) versus novel stimuli (i.e., innocent suspects or fillers). The greater the distance between these means, the greater the ability of the eyewitness to discriminate between target and non-target. For instance, increasing target memory strength will move the target distribution away from the non-target distribution, thereby increasing discriminability. In DFT terms, the easier it is for an eyewitness to detect facial information diagnostic of suspect guilt (i.e., diagnostic of the stimulus being a target rather than a non-target), the more likely the target distribution will be separated from the non-target distribution, thereby enhancing discriminability (Wixted and Mickes 2014). The theoretical motivations behind DFT have led researchers to derive and test predictions on important eyewitness issues such as why showups (presenting a single suspect as an ID procedure) yield lower discriminability compared to lineups (surrounding a suspect with known-innocent fillers who match the perpetrator’s description) (Colloff and Wixted 2019) and why description-matched fillers should be preferred to suspect-matched fillers (Carlson et al. 2019). Whereas the outcome of theoretical discriminability mainly concerns the development of theories, these theories are often tested by measuring differences in empirical discriminability.

Unlike theoretical discriminability, empirical discriminability is not constrained by a particular theory and therefore it does not rely on the assumptions of a specified model or distribution (Wixted and Mickes 2018). Within an eyewitness ID paradigm, empirical discriminability is the ability of a group of eyewitnesses to appropriately assign guilt or innocence to suspects, and is measured with the partial Area Under the Curve (pAUC). When eyewitnesses are better at distinguishing between who is innocent and who is guilty then empirical discriminability will be enhanced (i.e., a higher pAUC). Previous researchers (e.g., Carlson et al. 2019; Colloff and Wixted 2019; Wooten et al. 2020) have tested DFT using empirical discriminability, finding better performance for conditions in which witnesses are more readily able to notice diagnostic information and discount non-diagnostic information (e.g., a fair simultaneous lineup), compared to conditions where this process is more difficult (e.g., a showup, a sequential lineup). For example, Wooten et al. (2020) found that surrounding a suspect with as few as two fillers resulted in a higher pAUC than a showup. Following several of these studies, our goal is also to test DFT predictions concerning theoretical discriminability with ROC analysis, which reveals differences in empirical discriminability. The latter is of more applied interest than is theoretical discriminability (Wixted and Mickes 2012), and measures of theoretical and empirical discriminability typically agree (Wixted and Mickes 2018).

### Diagnostic Feature-Detection Theory

Wixted and Mickes (2014) developed DFT to explain why simultaneous lineups produce better discriminability compared to other types of ID procedures that present faces in isolation (e.g., showups, sequential lineups). The structure of a simultaneous lineup facilitates a comparison process that allows the witness to attend to diagnostic information. Presenting objects or faces simultaneously bolsters the discrimination process because differences are easier to notice and evaluate, which has been supported by research outside the realm of eyewitness ID (e.g., Gibson 1969; Mundy et al. 2007). DFT suggests that witnesses will compare/contrast faces when making an ID decision, in order to determine the most likely target. If a face is presented in isolation, the eyewitness will have a more difficult time distinguishing diagnostic from non-diagnostic facial information and may attribute too much weight to non-diagnostic information when making their ID decision. For example, if an eyewitness reports that the perpetrator was a Caucasian man with a long beard and is later presented with a long-bearded Caucasian suspect as a showup, the witness could ID him simply because these aspects match with their memory, even though they would match whether the suspect is innocent or guilty. However, if that same suspect was placed in a fair simultaneous lineup containing other Caucasian men with long beards, DFT predicts that the witness will discount the beard (and race) as non-diagnostic information and focus instead on facial characteristics not shared by all lineup members (i.e., diagnostic information).

Table 1 replicates the original DFT table from Wixted and Mickes (2014). A hypothetical eyewitness gleans information from one (showup) or more (lineup) individuals and compares this information to their memory for the perpetrator. On the far right is the overall memory match strength in the form of discriminability between innocent suspect (and fillers for the lineup) and guilty suspect:

$$d_{a} = \frac{{\mu_{{{\text{guilty}}}} - \mu_{{{\text{innocent}}}} }}{{\sqrt {(\sigma_{{{\text{guilty}}}}^{2} + \sigma_{{{\text{innocent}}}}^{2} )/2} }}$$

**Table 1:**
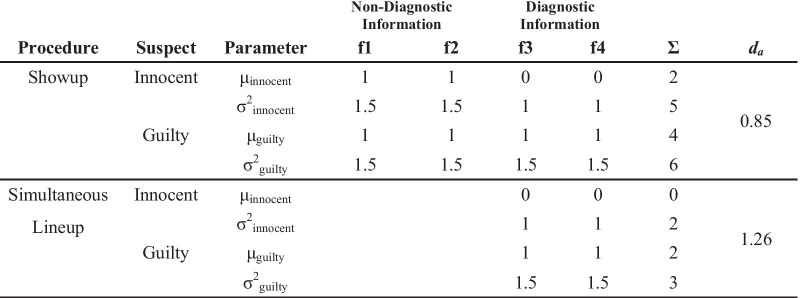
Diagnostic feature-detection theory modified from Table 1 of Wixted and Mickes (2014)

Non-diagnostic information is represented in the left-center two columns (f1 and f2), and diagnostic information is represented in the right-center two columns (f3 and f4). It is important to note that DFT is agnostic regarding what exactly the so-called features represent. Wixted and Mickes used examples such as face shape and size of eyes. However, any aspect of facial information, be it featural, holistic or configural, can be substituted in these columns. What is important is whether or not the information is diagnostic of suspect guilt (i.e., differs between the guilty and innocent suspect). Notice the blank areas in this table (f1 and f2, simultaneous lineup), which represent non-diagnostic information that is being discounted or unweighted in the eyewitness decision-making process. The idea in this example is that everyone in the lineup is of the same race and age, so the eyewitness discounts those aspects and focuses instead on diagnostic information such as face shape and size of eyes. This differential weighting of diagnostic over non-diagnostic information is what drives discriminability.

This original table from Wixted and Mickes (2014) demonstrates how removing non-diagnostic information from consideration increases discriminability, which has been supported empirically by showing that fair simultaneous lineups typically yield higher discriminability than do showups (e.g., Gronlund et al. 2012; Key et al. 2015; Wetmore et al. 2015b). More specifically, there are two predictions here: removing non-diagnostic information from consideration increases discriminability for lineups and also for showups. Both of these predictions have been separately supported in the literature. First, fair simultaneous lineups yield higher discriminability than biased simultaneous lineups (e.g., Colloff et al. 2016,2017; Flowe et al. 2014). Second, two studies have shown with just showups that discriminability can be increased by discounting non-diagnostic information. Colloff et al. (2018) presented participants with a target with a distinctive feature (e.g., a black eye) and later tested them with a guilty or innocent suspect as a showup. The distinctive feature remained on the suspect or was covered with a black rectangle. Being that the distinctive feature was non-diagnostic of suspect guilt (as both innocent and guilty suspect had the same feature), covering it increased discriminability. Colloff and Wixted (2019) also increased discriminability for showups by eliminating non-diagnostic information from consideration. They created a modified showup procedure that presented a suspect (guilty or innocent) with fillers, but these fillers could not be chosen (therefore it was not really a lineup). The authors argued that surrounding the suspect with similar faces allowed participants to determine facial information that was diagnostic versus non-diagnostic of guilt, thereby boosting discriminability beyond a typical showup.

Turning back to Table 1, there are three additional ways to change discriminability that have not been addressed in the literature: (a) adding non-diagnostic information, (b) removing diagnostic information, and (c) adding diagnostic information. First, DFT predicts that adding non-diagnostic information will reduce discriminability for the showup more so than the lineup, as this information is more likely to be discounted when eyewitnesses notice that it is shared among all lineup members. As described below, we added non-diagnostic information by presenting only the internal region of a target face at encoding and then adding the external region at test. For example, a perpetrator could commit a crime while wearing a hoodie that covers his hair and ears, but police may decide to present him to the eyewitness later with no hoodie, either in the form of a showup or lineup. In this scenario, DFT predicts that an eyewitness viewing a lineup would see how all lineup members share similar external features (assuming a fair lineup in which police do not want the suspect to stand out), and thereby discount^2^ the external features and focus instead on the internal features. In other words, it predicts that the eyewitness would evaluate the lineup in much the same way they would if the police had each lineup member wear a hoodie. In contrast, DFT predicts that showup performance would be particularly harmed by the added external features because the eyewitness cannot see that several individuals share similar external features and that, therefore, they are not helpful in determining guilt. In other words, it predicts that the eyewitness would perform better if the suspect was presented with a hoodie in the showup because then they are restricted from weighting external features in the decision-making process. We will test both of these predictions, calling the first the *Lineup with Non-Diagnostic Features Added Prediction* (i.e., similar discriminability whether external features are present or absent in a lineup) and the second the *Showup with Non-Diagnostic Features Added Prediction* (significantly reduced discriminability when external features are added to a showup).

**Table 2:**
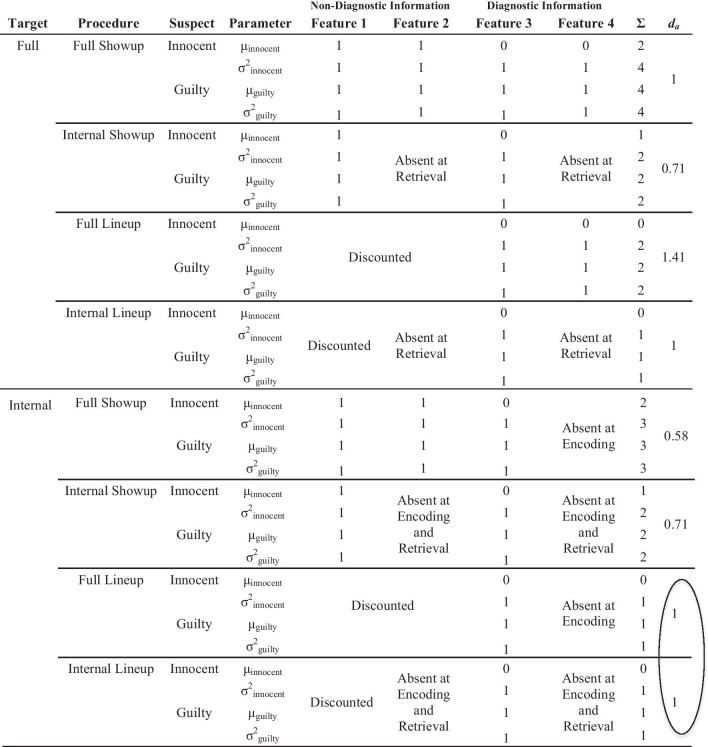
Diagnostic feature-detection theory predictions when manipulating the presence of diagnostic and non-diagnostic facial information at encoding and retrieval

Features 1 and 3 come from the internal face region and Features 2 and 4 come from the external face region. For simplicity, *Discounted* means that a Feature is completely eliminated from consideration, which assumes optimal decision-making. This is not an inherent assumption of DFT (because people are not perfectly optimal), but we apply it here for illustrative purposes. Most of these simple effects (i.e., discriminability comparisons in the right column) come from encoding specificity, with conditions that match encoding with retrieval being superior to mismatch conditions. However, the two circled discriminability values represent an important DFT prediction. They do not need to be equal, and Full Lineup could actually have somewhat lower discriminability due to adding noise (i.e., the external region of the face; e.g., Leder and Carbon 2005), but the difference between Internal-Full and Internal-Internal Lineup should be less than the difference between Internal-Full and Internal-Internal Showup. Both should be in the direction predicted by encoding specificity (i.e., Internal-Internal > Internal-Full), but for lineups, DFT predicts that eyewitnesses will notice shared qualities of these added features (e.g., they all have short dark hair) and therefore will discount these features to an extent not possible for a showup. This boosts discriminability for Full Lineups, but not Full Showups, thereby bringing the Internal-Full Lineup discriminability closer to Internal-Internal Lineup

The second way to change discriminability that has not been addressed in the literature is removing diagnostic information, which should lower discriminability for both showups and lineups. In our experiment, this is possible by presenting a full-face target at encoding and then at test presenting only the internal region of the face, either as a showup or in a lineup (with only internal face regions as fillers). Unlike the hoodie example above, we can think of no real-world example of this scenario and we included this manipulation for theoretical reasons only. Why would police intentionally hide part of the suspect’s face during an ID procedure, if it was visible during the crime and has not changed? We will call the first prediction the *Showup with Diagnostic Features Removed Prediction* (i.e., reduced discriminability when part of the face is hidden/removed in a showup) and *Lineup with Diagnostic Features Removed Prediction* (i.e., reduced discriminability when the same part of all lineup members is hidden/removed).

It is important to note that these DFT predictions also come from encoding specificity (Tulving and Thompson 1973). Whenever there is a change between encoding and retrieval, encoding specificity predicts a decline in performance, compared to when the specific nature of the encoded stimulus is re-presented at test. It predicts reduced performance when facial features are added or removed between encoding and test, compared to when the facial information remains the same between encoding and test. However, the *Lineup with Non-Diagnostic Features Added Prediction* modifies this prediction due to the DFT process of discounting non-diagnostic information. We still expect that the match condition (seeing internal face region at both encoding and retrieval) could outperform the mismatch condition (encoding internal region of face then tested with full faces), but, critically, we expect this difference to be smaller (and possibly nonsignificant) for lineups compared to showups. As described further below, this prediction is our most direct novel test of DFT. If there is a DFT process of discounting shared non-diagnostic information (which is only possible in lineups, not showups), this should reduce the encoding specificity effect of reduced performance (i.e., discriminability) for mismatch compared to match conditions, for lineups more so than showups.

The third and final method of changing discriminability within the context of DFT is adding diagnostic information, which should increase discriminability for both showups and lineups. However, we are not sure that this is possible in the real world and could not determine how to test it. How could police add diagnostic information to a suspect to make him an even better match to memory? We touch on one possibility in the Discussion as a future direction, but for the present study we did not attempt to test this.

### The Present Study

**Fig. 1:**
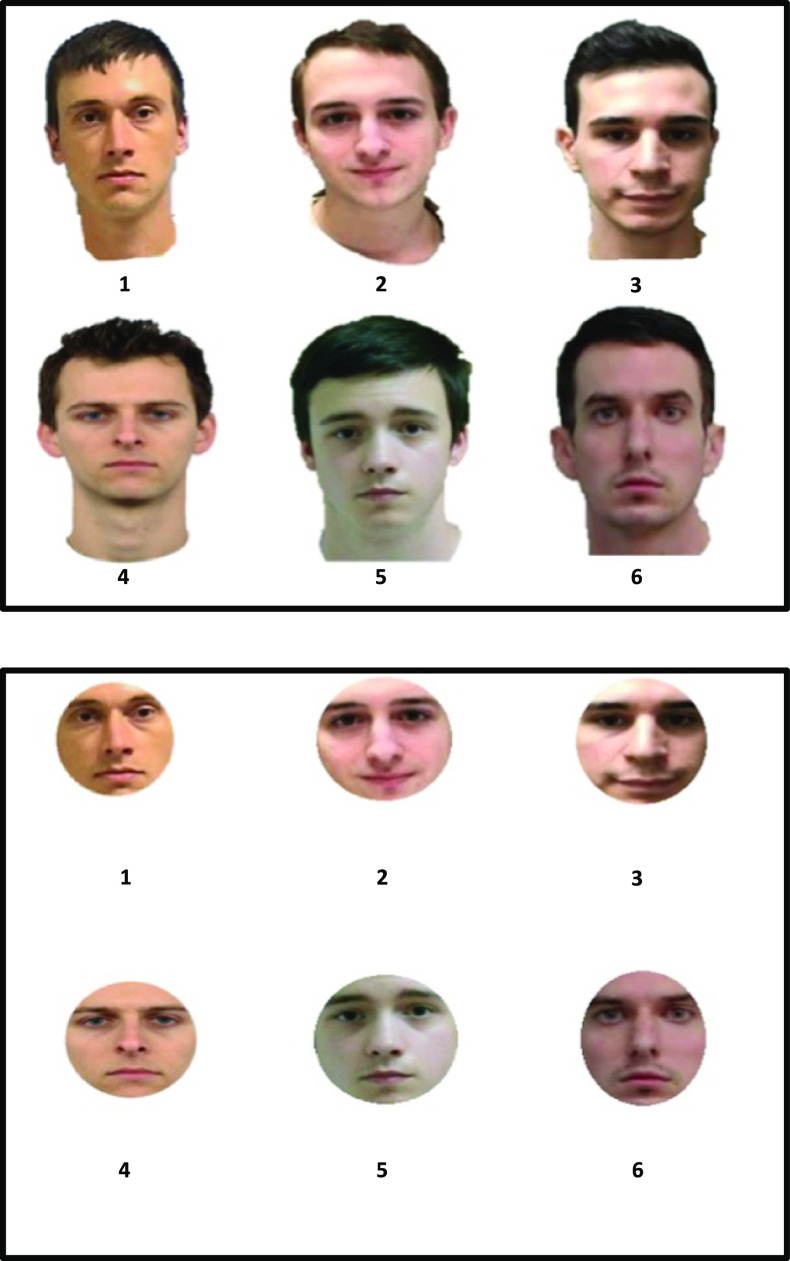
Top panel is an example full face lineup with Target 1 in Position 4. The bottom panel is the same lineup with the external facial areas removed. The internal faces were presented as the same size and position as they are in the full faces

Table 2 portrays our approach^3^ to testing these DFT predictions. Carrying over from Table 1 are the same two columns in the left-center depicting non-diagnostic information and two columns in the right-center depicting diagnostic information. As noted above, though we continue with the “feature” label here, the model is agnostic regarding whether diagnostic and non-diagnostic information is featural, configural, or holistic. We split the lineup and showup rows into full versus internal showups and lineups to illustrate our retrieval manipulations, and we added a column at the far left to represent our encoding manipulation. By requiring half of our participants to encode a partial-face target (heretofore called an *internal face*; see Fig. 1), this allowed us to test the predictions concerning *adding* non-diagnostic information because we could present a full face (showup) or full faces (lineup) at test. The same logic could not be applied to adding diagnostic information because, if the information is not in memory, how could it become diagnostic? This is why we had no predictions specific to this issue of potentially adding diagnostic information. However, we could certainly *remove* diagnostic information to test other predictions, by requiring other participants to encode a full target face and then be tested on an internal face (showup) or internal faces (lineup). Moreover, by factorially investigating full versus internal faces at both encoding and retrieval, as noted above we were also able to test encoding specificity predictions (Tulving and Thompson 1973). Match conditions (Full-Full and Internal-Internal) should generally yield higher discriminability than mismatch conditions (Full-Internal, Internal-Full).

When interpreting Table 2, it is important to note that Features 1 and 3 are based on the internal region of the face, whereas Features 2 and 4 come from the external region. We make no claims about the relative importance of internal versus external facial information. Rather, we are simply allowing the model, in the form of Table 2, to represent our methods for testing our hypotheses (i.e., removing diagnostic information or adding non-diagnostic information). Our predictions do not change if more features are added to the table.

There are several areas in Table 2 without memory strength values, representing information that is either discounted in the eyewitness decision-making process or was simply not present (at encoding and/or retrieval). Table 1 leaves these areas blank, but in Table 2 we provide more detail about these areas based on our manipulations. There is no difference between information that is discounted versus absent in the calculation of *d*_*a*_, but we think it is important to illustrate this distinction. We begin with the top half of Table 2 (full targets). When the target is encoded as a full face, non-diagnostic and diagnostic information can be removed at retrieval, as is the case for Features 2 and 4 (i.e., the external region of the face) in the internal showup and lineup conditions. This information is removed from the face (showup) or faces (lineup), and therefore cannot be appropriately or inappropriately given weight in an ID decision. An additional process occurs in lineups due to the presence of other faces sharing non-diagnostic facial information. For the full lineup, this information is discounted based on a DFT process (see also in Table 1). However, for the internal lineup, there are two separate reasons why non-diagnostic information is not included in the aggregate memory strength value on the right. Feature 1 is still discounted, as it is present in the form of internal facial information that is shared by all lineup members (e.g., they are all white males with no facial hair). Features 2 and 4, in contrast, came from the external area of the face, which is absent at retrieval.

Moving now to the bottom half of Table 2, we consider internal faces at encoding, which allowed us to add non-diagnostic information at retrieval, in the form of adding the external region of the face(s). We will begin with the implications for showups, followed by lineups. Starting with the full showup, Feature 4 is absent at encoding, and we argue that adding the external region of the face does not add diagnostic information (as it is not in memory), but does add non-diagnostic information (i.e., Feature 2, but not Feature 4, has memory strength values). In other words, participants may have difficulty ignoring the external region of the face that is now present along with the internal region. Due to holistic face processing, it is difficult for participants to ignore part of a test face that they are not being tested on. For example, Leder and Carbon (2005) presented participants with an individual facial feature (e.g., eyes, nose) and later tested with facial features or full faces. They found reduced accuracy when testing with full faces as opposed to features, indicating that participants could not ignore the rest of the test face, even though they were only being tested on, say, the nose. Another way to describe this phenomenon is simply that adding noise to a retrieval cue should reduce discriminability (e.g., Kent et al. 2018; Starns and Ratcliff 2014). We expect a similar process at work when we present only the internal region of the face at encoding and then add the external region at test (i.e., Internal-Full Showup). Next in Table 2 is the Internal-Internal showup, which is a simpler story: Both Features 2 and 4 (both from the external region of the face) are absent at both encoding and retrieval and can therefore have no memory strength values.

We turn now to the Internal-Full lineup from the bottom half of Table 2. Just like the Internal-Full showup, none of the external facial region can be considered diagnostic of suspect guilt, as it is not in memory. This is indicated in the right of Table 2 (Feature 4), which states *Absent at Encoding*. If the external region of the face carries no diagnostic information, then it must be non-diagnostic of guilt, which is represented by Feature 2. DFT includes a discounting process for shared non-diagnostic information, which for the sake of simplicity we portray in Table 2 as an all-or-none process, such that Feature 2 carries full decision weight for the Internal-Full showup but is fully discounted for the Internal-Full lineup. For example, after encoding the internal face region of a white male face, some participants see a lineup filled with full faces, all of which are white males with short dark hair. In this example, white and male are included in Feature 1, as these qualities are identifiable from the internal face region, and dark hair is in Feature 2, as it is identifiable only from the external face region. In our conceptualization of DFT, both of these Features will be discounted in the lineup because they are shared by all lineup members.^4^

Last in Table 2 is the internal lineup. Like the internal showup, Features 2 and 4 (i.e., the external region of the face) are absent at encoding and retrieval, therefore carrying no memory strength. Feature 1, in contrast, comes from the internal region, which is always present. However, just like the full lineup, it is shared by all lineup members and therefore can be discounted via a DFT process as non-diagnostic of suspect guilt.

The real-world representation of our encoding manipulation is perpetrators with disguises revealing only part of their face to eyewitnesses, and the closest analog is a perpetrator wearing a hoodie. There are several examples of innocent men sent to prison after eyewitnesses mistakenly identified them after seeing a crime in which a perpetrator wore a disguise (e.g., a ski mask: https://innocenceproject.org/cases/travis-hayes; a hoodie: https://painnocence.org/dontiapatterson). As for our presentation of full versus internal faces in showups and lineups, this is analogous to police determining how to present a suspect to eyewitnesses when the perpetrator was disguised. Very little research has investigated this issue, but Manley, Chan, and Wells (2018) found that if the perpetrator wore a ski mask, it is best for police to present a lineup in which all members also have a ski mask (see also Wetmore et al. 2015a). We will seek to replicate this finding in the form of internal face at encoding, followed by internal faces in a lineup. We will also determine whether or not this effect is present for showups.

### Predictions

Table 3 lists our predictions based on Table 2, and we will describe them in detail here. We will begin by describing our four main effect predictions, starting with two that, though they can be derived from Table 2, do not necessarily rely upon a DFT process. First, encoding a full target face should yield higher discriminability than encoding only internal facial information, which is based on the importance of external facial information for processing unfamiliar faces (e.g., Bonner et al. 2003; Frowd et al. 2007; Young et al. 1985). Second, encoding specificity (Tulving and Thompson 1973) and transfer-appropriate processing (Morris et al. 1977) predict that our match conditions (Full-Full and Internal-Internal) will outperform our mismatch conditions (Full-Internal and Internal-Full) overall. Third, we seek to replicate the commonly supported DFT prediction that fair simultaneous lineups will yield higher discriminability than showups. Fourth, DFT also predicts that full faces (i.e., more diagnostic facial information) will yield higher discriminability than internal faces at test, collapsed over the encoding manipulation and showups/lineups. This can be seen by averaging the discriminability estimates from all Full Showup and Full Lineup conditions from Table 2 ([1 + 1.41 + 0.58 + 1]/4 = 1), which exceeds the average of the discriminability estimates from all Internal Showup and Internal Lineup conditions ([0.71 + 1 + 0.71 + 1]/4 = 0.86). This effect should remain in the form of a simple effect for showups and lineups separately as well (two predictions that also come from Table 2).

**Table 3:** Predictions derived from DFT as represented in Table 2

	Discriminability prediction	Theoretical basis	Figure
Main effects	1) Full target > internal target	Face processing literature	2
	2) Match > mismatch	Encoding specificity	3
	3) Lineups > showups	DFT	4
	4) Full face(s) at retrieval > internal face(s) at retrieval	DFT	5
Simple effects	5) Full-full > full-internal	Encoding specificity	6
	6) Internal-internal > internal-full	Encoding specificity	6
	7) Internal-internal showup > internal-full showup	Encoding specificity	7
	8) Full-full showup > full-internal showup	Encoding specificity	7
	9) Full-full lineup > full-internal lineup	Encoding specificity	8
	*10) Internal-internal lineup ≥ internal-full lineup	DFT	8

DFT = Diagnostic feature-detection theory; *The ≥ symbol here represents the fact that we expect these two conditions to be either equivalent or with a small discriminability advantage for Internal-Internal. However, critically, we expect this difference (if significant), to be weaker than for the other match versus mismatch predictions. This expectation is due to an assumed DFT process of discounting of non-diagnostic information, which should occur for lineups and not for showups. According to DFT, discounting of non-diagnostic information in lineups boosts discriminability, which we expect to either bring Internal-Full Lineup up to the level of Internal-Internal Lineup, or at least closer than for showups

There are several additional simple effects that come from Table 2, based on interactions between the type of face encoded (full versus internal) and the type of face tested (full versus internal). Interestingly, DFT and encoding specificity converge upon the same predictions: (a) If a full face is encoded, discriminability will be higher for full face(s) compared to internal face(s) at test (collapsed over showups and lineups), (b) if an internal face is encoded, discriminability will be higher for internal face(s) compared to full face(s) at test (collapsed over showups and lineups), (c) if an internal face is encoded, discriminability will be higher for an internal showup compared to a full showup (*Showup with Non-Diagnostic Features Added Prediction*), (d) if an internal face is encoded, discriminability will be (slightly) higher for an internal lineup compared to a full lineup (*Lineup with Non-Diagnostic Features Added Prediction*), (e) if a full face is encoded, discriminability will be higher for a full showup compared to an internal showup (*Showup with Diagnostic Features Removed Prediction*), and (f) if a full face is encoded, discriminability will be higher for a full lineup compared to an internal lineup (*Lineup with Diagnostic Features Removed Prediction*).

However, as described above, we expect that the *Showup with Non-Diagnostic Features Added Prediction* will result in a larger discriminability difference (Internal-Internal Showup > Internal-Full Showup) than the *Lineup with Non-Diagnostic Features Added Prediction*. As shown in Table 2, DFT predicts equivalent performance between internal and full lineups in this case. This is an extreme case according to which an eyewitness is discounting non-diagnostic information 100% from a lineup, but we acknowledge that this optimality is unlikely in real-world eyewitnesses. Rather, as we explained above, it is likely that discriminability will be somewhat lower for Internal-Full Lineups compared to Internal-Internal Lineups, but we illustrate it as equivalent in Table 2 to emphasize the DFT process of discounting non-diagnostic information from lineups more so than showups. In essence, adding facial information at test that was not encoded should reduce performance, but this effect should be more pronounced in showups compared to lineups.

In sum, we have 10 predictions in the form of four main effects and six simple effects (see Table 3). One prediction is based on the face processing literature, six are based on encoding specificity, and three are based on DFT.

## Method

### Participants

Based on recent eyewitness ID studies applying ROC analysis to lineup data (Colloff et al. 2016; Wooten et al. 2020), we sought at least 1000 participants per cell. This might sound like an unusually large number of participants, but each was tested only once, in keeping with the majority of the eyewitness ID literature. Moreover, ROC analysis requires suspect IDs only, and these are broken down across confidence levels. Therefore, much of the data (e.g., filler IDs, rejections) lie outside of our primary analysis. Our full design (described below) involves 16 cells, so we needed at least 16,000 participants. However, we decided to collect more than this minimum amount of data because, based on our prior studies with SurveyMonkey, we expected to drop at least 10% of participants for various reasons (e.g., not completing the study, failing attention check). As a result of these considerations, we collected data from a nationwide sample of 20,604 participants via SurveyMonkey. We were left with 19,414 for analysis after dropping incompletes and failed attention checks, or an average of 1213 per cell. See Table 4 for demographics.

**Table 4:** Demographic information from our nationwide SurveyMonkey sample

	Nationwide SurveyMonkey sample
Sex	
Male	9,012
Female	10,402
Age	
18–29	5,225
30–44	4,351
45–60	6,796
Over 60	3,042
Ethnicity	
Black or African-American	1,298
White or Caucasian	13,359
Hispanic or Latino	1,769
Asian or Pacific Islander	1,620
American Indian or Alaskan Native	320
Other	353
Choose not to answer	695
*N*	19,414

### Stimuli

Four young male Caucasian targets were selected from the Radboud Face Database (Langner et al. 2010). We selected two images of each target: one angry expression to be presented at encoding and a neutral expression for the ID procedure (as all of our fillers also had a neutral expression). Fillers were selected from various prison databases (e.g., State of Kansas) and were selected based on their match to a given target. So, each target had its own pool of fillers, and from each pool we designated an innocent suspect based on being the best match to its respective target.

When constructing lineups, we used a same-fillers design (i.e., the same fillers were in both target-present [TP] and target-absent [TA] lineups). The simultaneous lineups were displayed in a 2 × 3 array, with the target and innocent suspect presented in either position^5^ 3 or 4. To ensure that we created fair lineups, we utilized a mock witness paradigm based on a modal description of the targets: “young Caucasian male with dark hair.” An independent sample of participants (*N* = 28) was asked to select the best match to the description for each lineup. We used these data to compute Tredoux’s *E’* (Tredoux 1998), a lineup fairness statistic that ranges from 1 (very biased) to 6 (very fair). All lineups used were fair according to this measure: Target 1 (TP 4.96, TA 5.17), Target 2 (TP 4.83, TA 4.58), Target 3 (TP 4.70, TA 5.24), Target 4 (TP 4.83, TA 5.32). For our internal face conditions, we used photoshop to remove the external region of both our encoding and test stimuli (Fig. 1).

### Design and Procedure

This experiment followed a 2 (target at encoding: full vs internal face) × 2 (test face(s): full vs internal) × 2 (ID procedure: showup vs simultaneous lineup) × 2 (target presence in ID procedure: TP vs TA) between-subjects factorial design, for a total of 16 experimental cells. Another way to describe the design involves encoding specificity: a match or mismatch between encoded and test faces. We had two match and two mismatch conditions: (a) full face at both encoding and test, (b) internal face at both encoding and test, (c) full face at encoding and internal face at test, (d) internal face at encoding and full face at test.

After providing informed consent, participants were instructed to “Pretend that you are about to witness a crime. On the next screen you will see the perpetrator's face for 5 s. Study it carefully.” They were then randomly assigned to one of the four targets for 5 s, presented as either full or internal face. Next, participants watched a 2-min distractor video about animals and then were asked a 4-item multiple-choice attention check question about the last animal they saw in the video. After completing the attention check, they were randomly assigned to a TP or TA showup or lineup, with either full or internal face(s). Just prior to their ID procedure, they were informed that the target may or may not be present. Those given a showup were instructed to identify the individual if he was the same person seen before. Those shown a lineup were instructed to either select a lineup member, labeled 1–6, if he was the person seen earlier, or select “none of the above.” After completing the ID procedure, participants were asked to rate their decision confidence on a 0–100% scale.^6^ Lastly, participants entered demographic information regarding their age, sex, and race.

## Results

Terminologically, *Full* will be used to represent the Full Face conditions (at encoding or test), and *Internal* will be used to represent the Internal Face conditions (at encoding or test). For example, the condition in which a Full Face target was encoded, but tested with the Internal region only (either showup or lineup), will be referred to as Full-Internal. Table 5 contains all ID decisions broken down by condition, and for the interested reader, “Appendix 1” contains separate analyses of TP and TA lineups to investigate the impact of our manipulations on correct IDs, filler IDs, and rejections from TP lineups, as well as innocent suspect IDs, filler IDs, and rejections from TA lineups. Being that our hypotheses involve discriminability, we focus on ROC analysis here.

**Table 5:** Counts and proportions for each decision category of the identification procedures

Encoded	Tested	Identification	Target-present			Target-absent		
Face	Face(s)	Procedure	Correct ID rate	Filler ID rate	Rejection rate	False ID rate	Filler ID rate	Rejection rate
Full	Full	Showup	.75 (929/1234)		.25 (305/1234)	.23 (275/1208)		.77 (933/1208)
		Lineup	.65 (793/1213)	.19 (236/1213)	.15 (184/1213)	.10 (123/1197)	.33 (392/1197)	.57 (682/1197)
	Internal	Showup	.47 (568/1203)		.53 (635/1203)	.18 (216/1209)		.82 (993/1209)
		Lineup	.44 (531/1218)	.25 (302/1218)	.32 (385/1218)	.13 (153/1213)	.40 (487/1213)	.47 (573/1213)
Internal	Full	Showup	.50 (615/1225)		.50 (610/1225)	.23 (284/1214)		.77 (930/1214)
		Lineup	.42 (511/1228)	.28 (344/1228)	.30 (373/1228)	.10 (121/1222)	.45 (550/1222)	.45 (551/1222)
	Internal	Showup	.60 (736/1219)		.40 (483/1219)	.22 (266/1209)		.78 (943/1209)
		Lineup	.46 (556/1201)	.30 (365/1201)	.23 (280/1201)	.12 (140/1201)	.42 (502/1201)	.47 (559/1201)

ID = Identification; Internal = internal region of the face only. Some proportions do not add up to 1.0 due to rounding error

### ROC Analysis

Although ROC analysis is a relatively new technique in its application to eyewitness ID (Wixted and Mickes 2012), it has a long history in applied fields including radiology and item recognition tasks (e.g., Macmillan and Creelman 2005). ROC analysis takes the ID decisions of individuals and jointly considers them with confidence judgments. This calculation allows for a numerical and graphical depiction of the performance of individuals across the entire range of confidence. With this analysis, one can assess both empirical discriminability and response bias independently (e.g., Gronlund et al. 2014; Rotello and Chen 2016; Wixted and Mickes 2012). As discussed above, empirical discriminability is measured by partial area under the curve (pAUC), a nonparametric measure that does not rely on theoretical assumptions (Wixted and Mickes 2018). When examining the performance of eyewitnesses in an ID task, it is most beneficial to determine the overall ability to sort guilty and innocent suspects into their appropriate categories, independent of their tendency to choose a suspect. This tendency to choose can be measured in terms of response bias, where eyewitnesses can be labeled as more conservative or more liberal in their likelihood of choosing. For example, a group of eyewitnesses making suspect IDs from fair simultaneous lineups could yield higher empirical discriminability than another group making suspect IDs from showups. However, the group viewing showups is likely to make more suspect IDs, representing a more liberal suspect choosing bias (e.g., Wetmore et al. 2015b).

Researchers utilize ROC analysis to separate empirical discriminability from suspect choosing bias to determine how each is impacting the performance of a group of participant-eyewitnesses, contrary to a confounding measure relied on more commonly in the past known as the diagnosticity ratio (DR; Gronlund et al. 2014; Wixted and Mickes 2012). The DR simply pits the observed correct IDs and false IDs against one another, without considering the impact that choosing rates might have on the overall performance of the eyewitness. The lack of response bias consideration confounds the data and can result in misleading conclusions regarding the ID performance of the eyewitnesses. Because of this issue, among others, ROC is the preferred method for analyzing ID performance in the eyewitness domain (National Research Council 2014), and its use has quickly accelerated over the last decade (e.g., Carlson and Carlson 2014; Carlson et al. 2019; Colloff and Wixted 2019; Gronlund et al. 2014; Jones et al. 2020; Meisters et al. 2018; Mickes et al. 2017; Wetmore et al. 2015b; Wooten et al. 2020).^7^ ROC analysis has been used to demonstrate several DFT predictions, such as the superiority of fair simultaneous lineups compared to showups (e.g., Wooten et al. 2020), sequential lineups (e.g., Carlson and Carlson 2014), and biased lineups (e.g., Wetmore et al. 2015b).

**Fig. 2:**
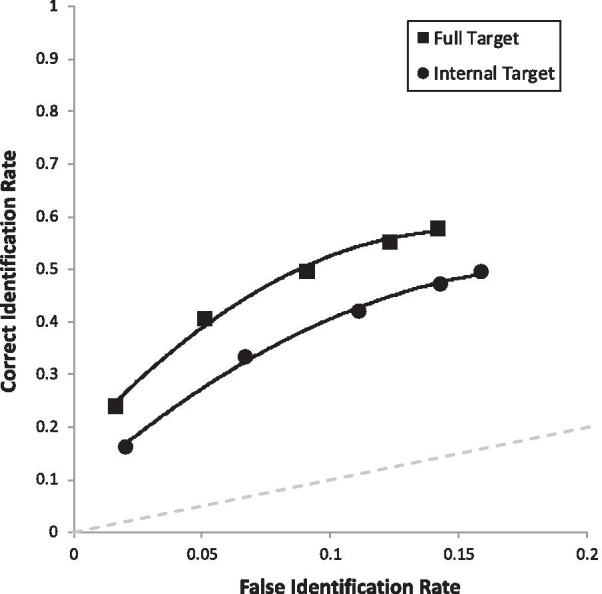
ROC curve for full targets versus internal targets (i.e., our encoding manipulation), collapsed over all other manipulations

Figure 2 depicts two ROC curves. Each curve is constructed based on a combination of correct ID rate (from TP lineups), false ID rate (from TA lineups), and the confidence in those IDs. Specifically, the far-right point on each curve represents the correct ID rate and false ID rate for that condition, regardless of confidence. The second point from the right simply drops out low-confidence IDs (0–30%), and the next point excludes more low-confidence IDs (0–50%), and so forth until the far-left point on each curve represents only high-confidence IDs (90–100%). Therefore, as curves extend further to the right, this represents more liberal suspect choosing, as more low-confidence IDs are included. The key measure is pAUC, which is a measure of empirical discriminability (e.g., Wixted and Mickes 2012; Gronlund et al. 2014), and pAUCs are compared with the statistic *D* = (pAUC1 − pAUC2)/*s*, where *s* is the standard error of the difference between the two pAUCs based on bootstrapping (see tutorial by Gronlund et al. 2014). With each pAUC are 95% CIs in brackets.

We conducted two sets of ROC analyses, first with false alarms from our designated innocent suspects, and again with all IDs from TA lineups (fillers and innocent suspects), divided by the nominal lineup size of six. Some of our designated innocent suspects were not chosen very often, based on the fact that we had fair lineups (see Tredoux’s *E’* values above) with fillers that were highly similar to the targets and innocent suspects. Therefore, we would argue that the second set of analyses is more meaningful, as it is not affected by the variability of innocent suspect choosing rates, and low overall innocent suspect choosing rates (see Table 5). Moreover, the most common approach in the eyewitness ROC literature is to utilize all IDs from fair TA lineups and divide by nominal lineup size (e.g., Humphries and Flowe 2015; Mickes 2015; Seale-Carlisle et al. 2019; Smith et al. [Bibr CR47][Bibr CR50]), and other researchers have done this even after designating innocent suspects, because they were chosen in unexpected ways (e.g., Carlson et al. 2016; Mansour 2020). As a result, we decided to report below only our analyses based on all IDs from TA lineups. However, the false ID rates reported in Table 5 are based on our designated innocent suspects, and later we apply signal detection analysis to these data. All patterns remained the same between the two sets of analyses.

Another issue is that, when comparing pAUCs, a decision must be made regarding specificity (1-FAR). In other words, when comparing two ROC curves, a cutoff point must be determined for the x-axis. See Fig. 4 for example. In replication of other studies finding more liberal suspect choosing from showups compared to fair simultaneous lineups (e.g., Key et al. 2017; Wetmore et al. 2015b), the showup curve extends further to the right than does the lineup curve. Therefore, the curves can be compared with two different specificities: (a) 1-max FAR of the longer curve, which includes all data from both curves, but requires extrapolating from the lineup curve; or (b) 1-max FAR of the shorter curve, which prevents any extrapolation but cuts off a region of the longer curve. We conducted both sets of analyses, which revealed the same pattern of results. Below we report the results based on option (a), as it does not exclude any data.

**Fig. 3:**
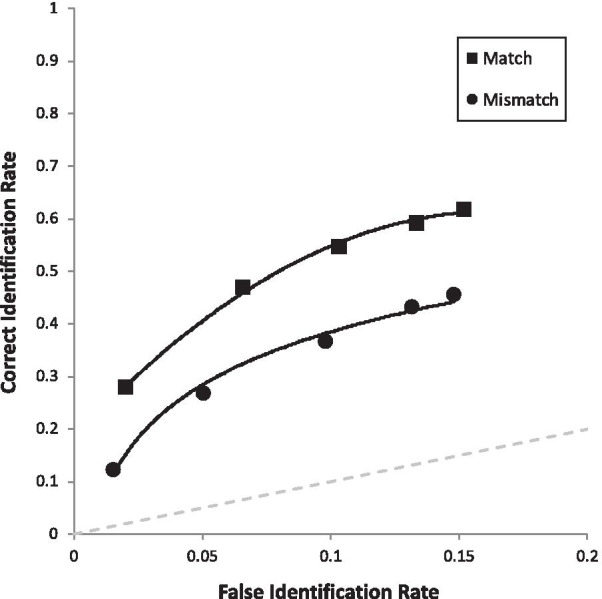
ROC curve for match (Full-Full and Internal-Internal) versus mismatch conditions (Full-Internal and Internal-Full), collapsed over all other manipulations

**Fig. 4:**
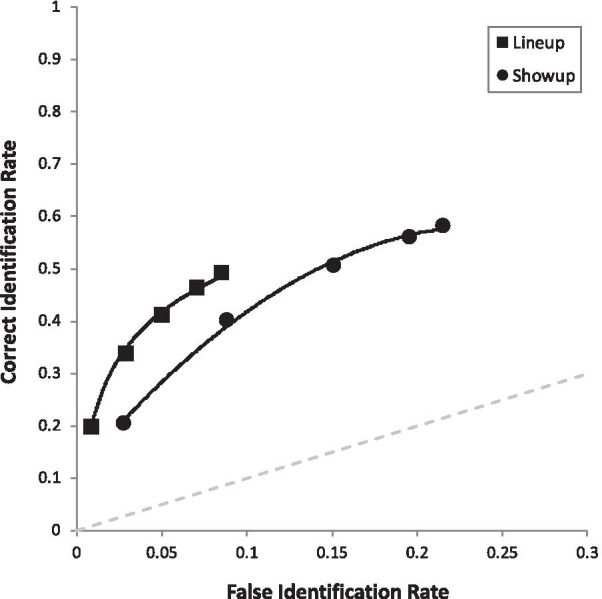
ROC curve for lineups versus showups, collapsed over all other manipulations

**Fig. 5:**
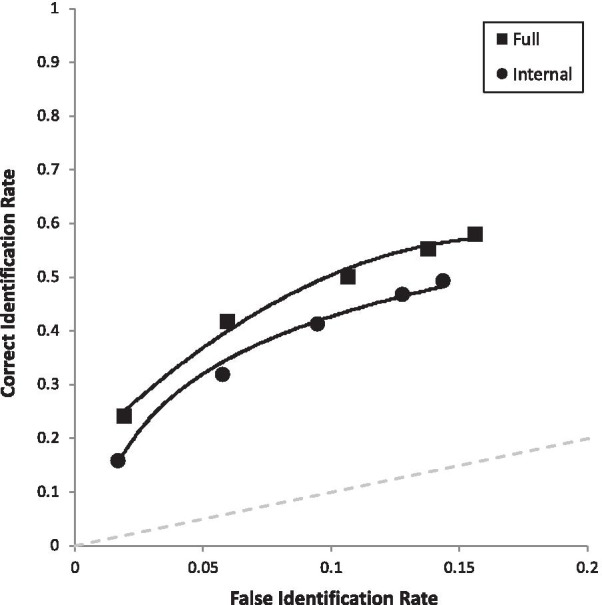
ROC curve for faces tested as full versus internal, collapsed over all other manipulations

Due to the fact that we had 10 predictions, we compared each *p* value with Bonferroni-corrected alpha of 0.05/10 = 0.005. We will describe our results in the same order as the hypotheses are laid out in the predictions section above (and Table 3), starting with the four main effects. First, in support of the face processing literature, full targets (pAUC = 0.070 [0.067, 0.072]) yielded higher discriminability than internal targets (pAUC = 0.052 [0.050, 0.055]), collapsed over all retrieval manipulations, *D* = 8.30, *p* < 0.001 (Fig. 2). Second, in support of encoding specificity, match conditions (Full-Full and Internal-Internal combined; pAUC = 0.072 [0.069, 0.075]) were superior to mismatch conditions (Full-Internal or Internal-Full combined; pAUC = 0.052 [0.050, 0.055]), *D* = 9.57, *p* < 0.001 (Fig. 3). Third, in support of DFT, we replicated the common advantage of simultaneous lineups (pAUC = 0.102 [0.098, 0.105]) over showups (pAUC = 0.077 [0.084, 0.092]), *D* = 5.52, *p* < 0.001 (Fig. 4). Fourth, also in support of DFT, full face(s) at retrieval (pAUC = 0.067 [0.064, 0.069]) yielded higher discriminability than internal face(s) at retrieval (pAUC = 0.055 [0.052, 0.058]), *D* = 5.44, *p* < 0.001 (Fig. 5).

**Fig. 6:**
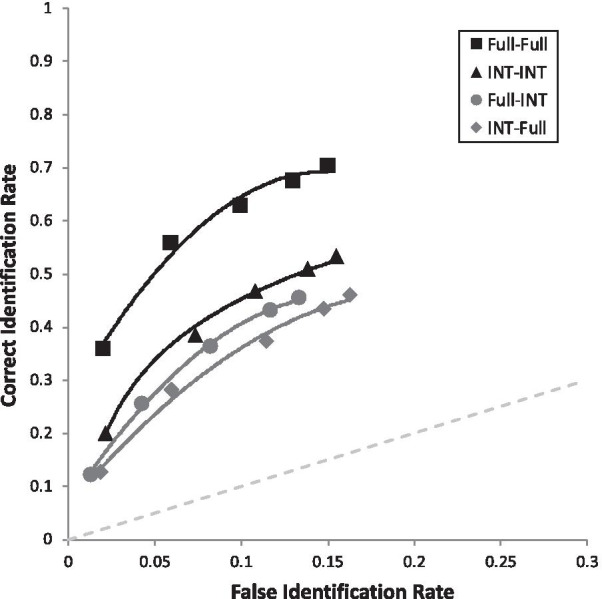
ROC curve for each match versus mismatch condition. Full = full face; INT = internal face only. For example, Full-INT represents a full face presented at encoding, followed later by internal face, collapsed over showups and lineups

Moving now to simple effects, we had six predictions. The first two were derived from both DFT (see Table 2) and encoding specificity. Figure 6 breaks the two ROC curves from Fig. 3 into four curves in order to test these two predictions. In support of both theories, when collapsed over lineups and showups, Full-Full (pAUC = 0.093 [0.089, 0.098] yielded higher discriminability than Full-Internal (pAUC = 0.057 [0.053, 0.061]), *D* = 12.04, *p* < 0.001, and Internal-Internal (pAUC = 0.064 [0.059, 0.068] increased discriminability compared to Internal-Full (pAUC = 051 [0.047, 0.055]), *D* = 4.37, *p* < 0.001.

**Fig. 7:**
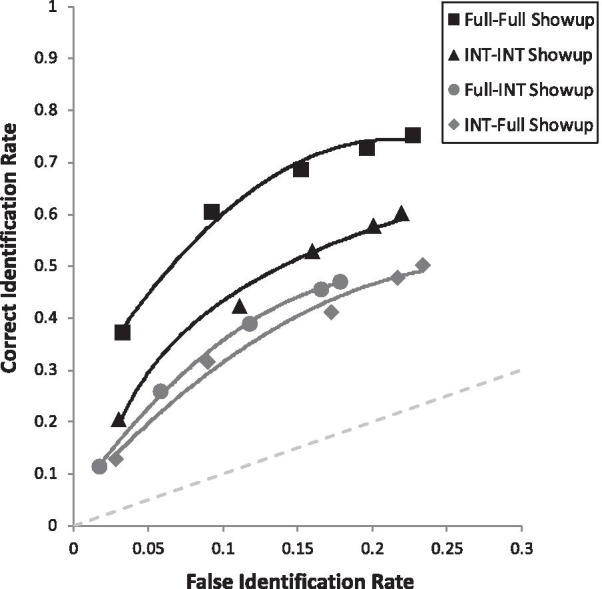
ROC curve for each showup condition broken down by encoded and test face type. Full = full face; INT = internal face only. For example, Full-INT represents a full face presented at encoding, followed later by internal face

**Fig. 8:**
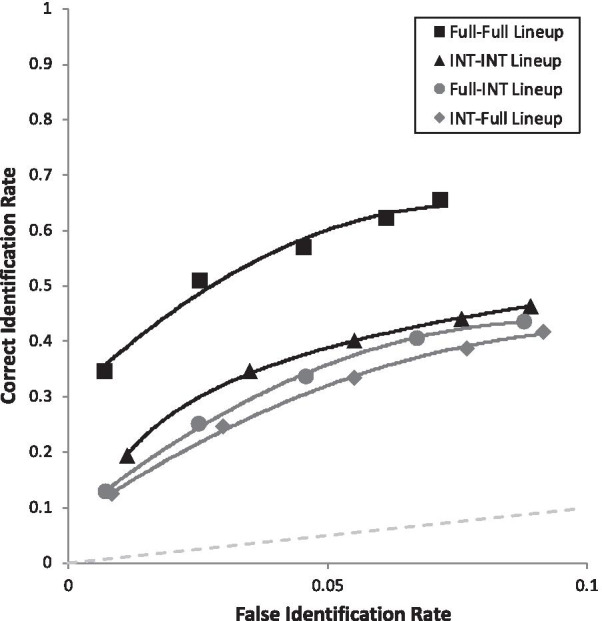
ROC curve for each lineup condition broken down by encoded and test face type. Full = full face; INT = internal face only. For example, Full-INT represents a full face presented at encoding, followed later by internal face

**Fig. 9:**
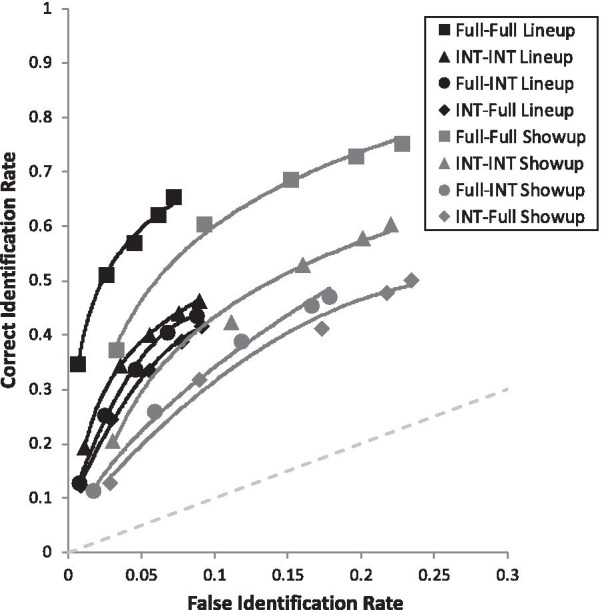
ROC curve for each condition represented in Table 2

Lastly are the simple effects that address our four primary DFT predictions. First, we supported the *Showup with Non-Diagnostic Features Added Prediction*, such that Internal-Internal Showup (pAUC = 0.098 [0.090, 0.106]) was superior to Internal-Full Showup (pAUC = 0.077 [0.069, 0.084]), *D* = 3.65, *p* < 0.001 (Fig. 7). Second, we supported the *Showup with Diagnostic Features Removed Prediction*, such that Full-Full Showup (pAUC = 0.138 [0.129, 0.146]) yielded higher discriminability than the Full-Internal Showup (0.088 [0.081, 0.096]), *D* = 8.63, *p* < 0.001 (Fig. 7). Third, we supported the *Lineup with Diagnostic Features Removed Prediction*, such that Full-Full Lineup (pAUC = 0.055 [0.051, 0.059]) was superior to Full-Internal Lineup (pAUC = 0.032 [0.028, 0.035]), *D* = 8.45, *p* < 0.001 (Fig. 8). Fourth, the *Lineup with Non-Diagnostic Features Added Prediction* was that Internal-Internal Lineups and Internal-Full Lineups would be equivalent or with a slight advantage for Internal-Internal Lineups, but encoding specificity predicts that Internal-Internal should be clearly superior to Internal-Full. We found that Internal-Internal Lineups yielded equivalent discriminability (pAUC = 0.035 [0.031, 0.038]) as Internal-Full Lineups (pAUC = 0.029 [0.026, 0.033]), *D* = 2.14, *p* = 0.033 (Fig. 8), in support of DFT. As a reminder, this is nonsignificant based on the Bonferroni-corrected alpha of 0.005. For the interested reader, Fig. 9 portrays all eight conditions from Table 2 together in one graph.

### SDT Analysis

To support our ROC analyses, we confirmed all differences among pAUCs with SDT analysis applied to correct and false ID rates from Table 5. In Table 6, we present the *d'* for each condition included in our 10 predictions, along with 95% inferential confidence intervals to allow evaluation of statistical significance. This table establishes two points: (a) the theoretical discriminability results match the empirical discriminability results, and (b) the results are the same regardless of how false ID rate is calculated, such that the results based on all IDs from TA lineups (i.e., the pAUCs) match those based on designated innocent suspects (i.e., the *d'*s). Note how the last two conditions listed in Table 6 yield virtually identical *d'*s, providing additional support for this DFT prediction.

**Table 6:** Estimates of theoretical discriminability and inferential confidence intervals (ICI) for each condition

Condition	*d'*	95% ICI
Full target	1.20	1.19–1.21
Internal target	0.95	0.95–0.96
Match	1.26	1.25–1.27
Mismatch	0.89	0.88–0.89
Lineups	1.20	1.19–1.21
Showups	0.97	0.96–0.98
Full face(s) at retrieval	1.18	1.17–1.19
Internal face(s) at retrieval	0.97	0.96–0.98
Full-full	1.50	1.48–1.52
Full-internal	0.90	0.88–0.92
Internal-internal	1.03	1.00–1.05
Internal-full	0.87	0.85–0.90
Internal-internal showup	0.84	0.80–0.88
Internal-full showup	0.74	0.71–0.78
Full-full showup	1.41	1.37–1.46
Full-internal showup	0.84	0.79–0.90
Full-full lineup	1.67	1.61–1.72
Full-internal lineup	0.98	0.93–1.02
Internal-internal lineup	1.07	1.01–1.13
Internal-full lineup	1.08	1.03–1.14

## Discussion

We conducted a high-powered experiment with a demographically diverse sample to test several predictions derived from diagnostic feature-detection theory (DFT; Wixted and Mickes 2014) and encoding specificity (Tulving and Thompson 1973). Essentially DFT boils down to the presence or absence of diagnostic and non-diagnostic facial information. Diagnostic information is that which differs between the guilty and the innocent (e.g., the perpetrator had blue eyes, but the innocent suspect and fillers have brown eyes); non-diagnostic information is shared by the innocent and the guilty (e.g., the perpetrator had a beard, and so too does the innocent suspect and all fillers). Researchers primarily have tested DFT by discounting non-diagnostic facial information, with four different approaches, comparing: (a) showups with fair simultaneous lineups (e.g., Wooten et al. 2020), (b) fair simultaneous lineups with fair sequential lineups (e.g., Carlson and Carlson 2014), (c) fair simultaneous lineups with biased simultaneous lineups (e.g., Colloff et al. 2016), and (d) showups with showups with non-diagnostic information removed or discounted (e.g., Colloff et al. 2018). These four methods discount non-diagnostic information in different ways, such as adding good fillers or explicitly covering a non-diagnostic feature.

In contrast, we tested DFT by adding non-diagnostic information and also by removing diagnostic information. Both were made possible by manipulating the presence of facial information (i.e., the external region of the face) at both encoding and at retrieval. Therefore, some participants could see more facial information at retrieval than they did at encoding (i.e., our Internal-Full conditions), and others could see less facial information at retrieval than they did at encoding (i.e., our Full-Internal conditions). As shown in Tables 2 and 3, we derived 10 predictions from DFT and encoding specificity (four main effects, and six simple effects based on interactions). Some of these serve to support DFT alone, and most serve to support DFT in combination with a well-supported theory from either the memory literature (encoding specificity) or the face processing literature (worse memory for faces without external features). With both a measure of empirical discriminability (pAUC) and a measure of theoretical discriminability (*d'*), we supported all of these predictions. One of the 10 predictions (Internal-Internal Lineup ≥ Internal-Full Lineup) represents a DFT prediction that qualifies the typical pattern expected based on encoding specificity (Internal-Internal Lineup > Internal-Full Lineup). According to DFT, there will be some degree of discounting of shared non-diagnostic information in a lineup that is not possible in a showup, and therefore, we expected that the typical match > mismatch pattern from encoding specificity would be either reduced or eliminated. The comparison of pAUCs revealed a reduced and nonsignificant match > mismatch effect here, which was not the case for any other match versus mismatch comparison (all *p*’s < 0.001). Moreover, the *d'*s were virtually identical.

This is arguably our most important finding, so we will expand our interpretation of it here. We presented an oversimplification of DFT assumptions in Table 2, including the discounting process for shared features. This is particularly relevant for the bottom half of the table, which is for conditions in which only the internal face region was encoded. In this case, there are two possibilities for the retrieval scenario: (a) present an internal face region again (Internal-Internal), or (b) add the external region to the internal region to present a full face (Internal-Full). Encoding specificity is straightforward in predicting Internal-Internal > Internal-Full. However, the DFT prediction is more complicated, especially when it comes to the Internal-Full condition. The first claim we make is that none of the external face information can be diagnostic of suspect guilt, as it was not seen before. This appears to be noncontroversial. So, the information from the external face region must be non-diagnostic of guilt. In this case, DFT states that *shared* information will be discounted, which is a scenario possible for a lineup, but not for a showup (as no information can be shared across lineup members in a showup). For the sake of simplicity in the table, we assumed 100% discounting of non-diagnostic information from the lineup and 0% discounting in the showup. This assumes optimality in the decision-making process, which is unlikely in real eyewitnesses. A more realistic scenario is that discriminability will be harmed by adding non-diagnostic facial information (e.g., Leder and Carbon 2005), which is akin to adding noise to a memory trace (e.g., Kent et al. 2018; Starns and Ratcliff 2014). This means that DFT, similar to encoding specificity, would predict lower discriminability for Internal-Full compared to Internal-Internal. However, the key to DFT is that, for lineups, non-diagnostic information is discounted more so than diagnostic information, and this difference is greater than for showups (as the discounting process applies to shared features only). Therefore, discriminability, even though reduced by the added noise, should be boosted somewhat by this discounting process. Critically, this should occur for lineups and not showups. In sum, this means that the advantage for Internal-Internal over Internal-Full (i.e., encoding specificity) should be greater for showups than for lineups. This is exactly what we found, as the pAUCs numerically supported the pattern of Internal-Internal > Internal-Full for lineups, but unlike all other pAUC comparisons (all *p*’s < 0.001), it was nonsignificant. Additionally, there was no difference in *d'* between these two conditions. We conclude from these patterns that there is evidence of a discounting process applied to added non-diagnostic information that is stronger for lineups than for showups, which supports DFT.

Our results fit well with recent studies testing other novel predictions from DFT (Carlson et al. 2019; Wooten et al. 2020). For example, Carlson and colleagues supported the prediction that lineups containing highly similar fillers would reduce discriminability compared to fair lineups containing moderately similar fillers. DFT requires a certain level of heterogeneity of facial information in order to allow discrimination between what is diagnostic versus non-diagnostic of guilt. If all fillers are almost clones (as Carlson et al. assessed with computer-generated faces in their E1) or if they all look too similar to each other and the suspect (e.g., with a match-to-target instead of a match-to-description filler selection strategy as in their E2), then the DFT process is harmed because there is less diagnostic information or its diagnosticity is more difficult to discern. Our results expand upon these findings by showing that the outright removal of diagnostic information (i.e., our Full-Internal conditions) also harms discriminability.

We encourage other researchers to further test DFT, or to present alternative quantitatively specified models against which DFT can compete. There is much fertile ground left to investigate, such as the aforementioned theoretical possibility of adding diagnostic information. As we noted before, we struggle to think of a real-world example of this, and it could also be difficult to execute in an experiment. One possibility is to present a blurry image of a target, and later testing with a crystal-clear image. This would be akin to a witness who views a perpetrator from a distance (e.g., Lockamyeir et al. 2020), and is later presented with a showup up close or a high-definition mugshot with fillers in a photo lineup. Another way of testing DFT is by comparing a showup with a fair simultaneous lineup and also a showup plus base rate information. It is possible that one component of the discriminability advantage of lineups over showups is that the fillers present in lineups provide information about how one’s memory for the perpetrator fits into the base rate of individuals in the population. For example, the perpetrator could have blue eyes and a beard, and eyewitnesses would be likely to choose a suspect with blue eyes and a beard presented as a showup. But what if they are reminded that many individuals in the population have blue eyes and a beard? Would this instruction simply make them less willing to make an ID (i.e., inducing conservative response bias)? Or could it also increase discriminability, akin to seeing fillers in a lineup who also have blue eyes and a beard? In other words, are fair simultaneous lineups superior to showups only because of a DFT-like process, or could it also come partly from this reduction of base rate neglect? Or could the DFT-like process (i.e., discounting non-diagnostic information and focusing instead on diagnostic information) be triggered just with the base rate instruction, without any need for fillers? These are just some of the empirical questions ready to be tested.

## Conclusions and Implications

It is important for researchers to test quantitatively specified theories in order to develop robust methods to help police get the most from eyewitnesses. DFT is shaping up to be a powerful theory of eyewitness decision-making. It can help police to not only understand the importance of presenting fair simultaneous lineups over showups, but also to understand what makes a lineup fair. It is well known that a suspect should not stand out (e.g., Colloff et al. 2016), but DFT is also helping researchers understand that fillers should not look too much like the suspect; therefore, a match to description approach may be best (Carlson et al. 2019). The present research expands these retrieval-based implications to include encoding circumstances as well. Our internal face conditions are akin to a perpetrator wearing a disguise such as a hoodie that covers up external facial characteristics. How should police construct an ID procedure in this instance? Yes, they should present a fair simultaneous lineup over a showup, but should everyone in the lineup wear a hoodie? Both encoding specificity and DFT suggest that they should, which is an excellent example of theory-driven research providing concrete recommendations for police procedures.

## Figures and Tables

**Fig. 10:**
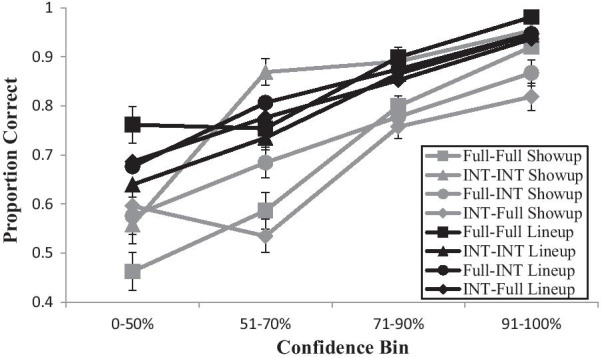
CAC curves for all conditions, with standard error bars. Full = full face; INT = internal features only

**Fig. 11:**
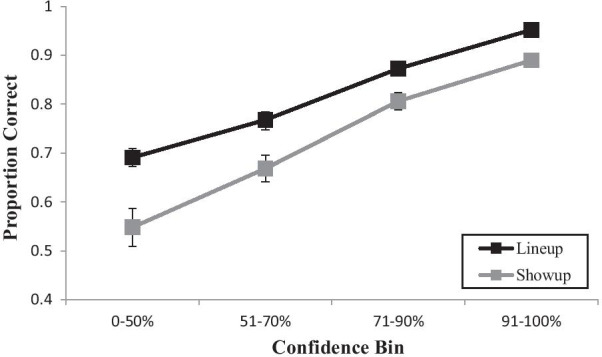
Overall CAC curve for lineups versus showups, with standard error bars

**Fig. 12:**
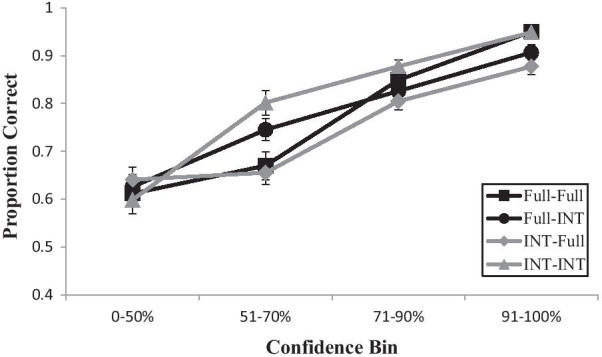
CAC curves across the four combinations of encoded versus tested face type (Full = full face; INT = internal features only), with standard error bars

**Fig. 13:**
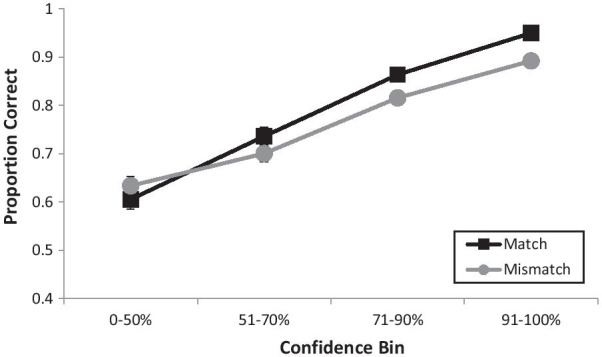
Overall CAC curve for match (Full-Full and Internal-Internal) versus mismatch conditions (Full-Internal and Internal-Full), with standard error bars

**Fig. 14:**
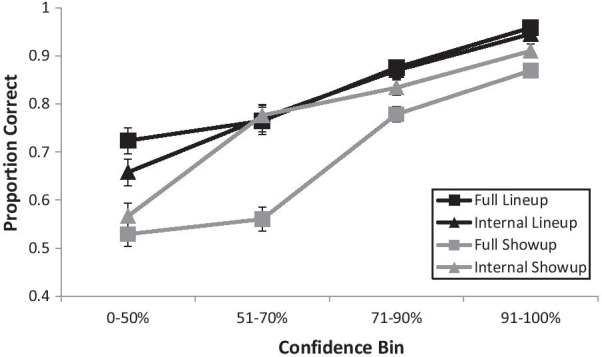
CAC curves across the four combinations of test face type (Full Face versus Internal Features only) and procedure (lineup versus showup), with standard error bars

## Data Availability

The dataFeature from this experiment is available from the first author on reasonable request.

## Appendix 1: Separate Analysis of Target-Present and Target-Absent Lineups

For both TP and TA data, we tested a binary logistic regression model with all manipulated variables except target presence: (a) encoded face type (Full vs. Internal), (b) tested face(s) type (Full vs. Internal), and (c) ID procedure (showup vs. lineup). The model also included the 3-way interaction of encoded face type X tested face(s) type X ID procedure, as well as the three included 2-way interactions: (a) encoded face type X tested face(s) type, (b) encoded face type X ID procedure, and (c) tested face(s) type X ID procedure. If a main effect or interaction was significant, we followed up with Chi-square analyses to investigate simple effects. We utilized an alpha level of 0.05 for all planned comparisons and applied a Bonferroni adjustment (alpha/# comparisons) for each measure (e.g., correct IDs from TP lineups, false IDs from TA lineups).

### TP Lineup Decisions

We will begin with correct IDs. There was a main effect of encoded face type (Wald (1) = 77.96, *p* < 0.001), test face type (Wald (1) = 84.64, *p* < 0.001), and ID procedure (Wald (1) = 86.07, *p* < 0.001). The effect of encoded face type is in the form of more correct IDs after encoding full compared to internal faces (*χ*^2^(1, *N* = 9741) = 67.97, *p* < 0.001, *ϕ* = 0.08), and this pattern occurred for both showups (*χ*^2^ (1, *N* = 4881) = 18.99, *p* < 0.001, *ϕ* = 0.06) and lineups (*χ*^2^ (1, *N* = 4860) = 53.96, *p* < 0.001, *ϕ* = 0.11). The effect of test face type is also in the form of more correct IDs for full compared to internal faces (*χ*^2^ (1, *N* = 9741) = 74.70, *p* < 0.001, *ϕ* = 0.09), and this pattern occurred for both showups (*χ*^2^ (1, *N* = 4881) = 40.22, *p* < 0.001, *ϕ* = 0.09) and lineups (*χ*^2^ (1, *N* = 4860) = 35.00, *p* < 0.001, *ϕ* = 0.09). The effect of ID procedure is in the form of more correct IDs for showups compared to lineups, *χ*^2^ (1, *N* = 9741) = 82.04, *p* < 0.001, *ϕ* = 0.09.

There was also an interaction between encoded and test face type (Wald (1) = 276.66, *p* < 0.001), and a marginally significant interaction between encoded face type and ID procedure (Wald (1) = 3.25, *p* = 0.07). There was no interaction between test face type and ID procedure, Wald (1) = 0.50, *p* = 0.48. Lastly, the 3-way interaction (encoded face type X test face type X ID procedure) was significant, Wald (1) = 10.89, *p* = 0.001.

As a result of these interactions, we next conducted several additional Chi-square analyses to test for simple effects. We start with showups versus lineups across the four combinations of encoded and test face types (Bonferroni-corrected alpha = 0.0125). Showups yielded more correct IDs than lineups in three of the four conditions: (a) Full-Full, *χ*^2^ (1, *N* = 2447) = 28.80, *p* < 0.001, *ϕ* = 0.11; (b) Full-Internal, *χ*^2^ (1, *N* = 2421) = 3.20, *p* = 0.08, *ϕ* = 0.04, (c) Internal-Full, *χ*^2^ (1, *N* = 2453) = 18.23, *p* < 0.001, *ϕ* = 0.09, and (d) Internal-Internal, *χ*^2^ (1, *N* = 2420) = 48.21, *p* < 0.001, *ϕ* = 0.14.

We now turn to the effect of encoding full versus internal faces across the four combinations of ID procedure and test face type: (a) more correct IDs for full over internal when tested with full showup, *χ*^2^ (1, *N* = 2459) = 165.49, *p* < 0.001, *ϕ* = 0.26; (b) more correct IDs for full over internal when tested with full lineup, *χ*^2^ (1, *N* = 2441) = 138.48, *p* < 0.001, *ϕ* = 0.24; (c) more correct IDs for internal over full when tested with internal showup, *χ*^2^ (1, *N* = 2422) = 42.21, *p* < 0.001, *ϕ* = 0.13; and (d) no difference between full and internal when tested with internal lineup, *χ*^2^ (1, *N* = 2419) = 1.78, *p* = 0.19, *ϕ* = 0.03. In sum, correct IDs were generally maximized when the test procedure matched encoding, particularly for Full-Full, but also for Internal-Internal (showups only). The exception is lineups containing internal faces.

We discovered a similar pattern when investigating the effect of testing with full versus internal faces across the four combinations of encoded face type and ID procedure, such that correct IDs generally increased when there was a match between encoding and test: (a) more correct IDs for full over internal showup when full target was encoded, *χ*^2^ (1, *N* = 2437) = 202.54, *p* < 0.001, *ϕ* = 0.29, (b) more correct IDs for full over internal lineup when full target was encoded, *χ*^2^ (1, *N* = 2431) = 116.24, *p* < 0.001, *ϕ* = 0.22, (c) more correct IDs for internal over full showup when internal target was encoded, *χ*^2^ (1, *N* = 2444) = 25.58, *p* < 0.001, *ϕ* = 0.10, and (d) marginally more correct IDs for internal over full lineup when internal target was encoded, *χ*^2^ (1, *N* = 2429) = 5.41, *p* = 0.02, *ϕ* = 0.05.

We conclude our TP analyses with a binary logistic regression analysis of filler IDs (1 = filler ID, 0 = any other lineup decision), which includes all of the same variables and tests for interactions as for correct IDs, except procedure, as showups have no filler IDs to analyze. There was a main effect of encoded face type (Wald (1) = 33.17, *p* < 0.001) and also tested face(s) type (Wald (1) = 10.15, *p* = 0.001). However, these two variables did not interact, Wald (1) = 2.32, *p* = 0.13. There were more filler IDs after encoding internal faces compared to full faces, *χ*^2^ (1, *N* = 4860) = 31.73, *p* < 0.001, *ϕ* = 0.08. There were also more filler IDs after being tested on internal faces compared to full faces, *χ*^2^ (1, *N* = 4860) = 9.26, *p* = 0.003, *ϕ* = 0.04.

### TA Lineup Decisions

We now turn to innocent suspect IDs from TA lineups. There was a main effect of ID procedure (Wald (1) = 193.97, *p* < 0.001), such that there were more false IDs for showups compared to lineups. There was no main effect of encoded face type (Wald (1) = 0.21, *p* = 0.65) or tested face(s) type (Wald (1) = 0.18, *p* = 0.67). The only significant interaction was between tested face(s) type and ID procedure, Wald (1) = 10.57, *p* = 0.001, but the interaction between encoded face type and ID procedure was marginally significant, Wald (1) = 2.80, *p* = 0.09. The 3-way interaction was not significant (Wald (1) = 0.87, *p* = 0.35), and there was no interaction between encoded and test face type (Wald (1) = 0.34, *p* = 0.56).

As a result of the interaction between tested face(s) type and ID procedure, we proceeded with Chi-squares to investigate simple effects (Bonferroni-corrected alpha = 0.0125). When comparing full and internal faces at test, there were (a) marginally more false IDs for full versus internal showup when a full target was encoded, *χ*^2^ (1, *N* = 2417) = 6.10, *p* = 0.014, *ϕ* = 0.05; (b) no difference in false IDs between full and internal showup when internal target was encoded, *χ*^2^ (1, *N* = 2423) = 0.59, *p* = 0.47, *ϕ* = 0.016, (c) no difference in false IDs between full and internal lineup when full target was encoded, *χ*^2^ (1, *N* = 2410) = 3.25, *p* = 0.07, *ϕ* = 0.037, and (d) no difference in false IDs between full and internal lineup when internal target was encoded, *χ*^2^ (1, *N* = 2423) = 2.13, *p* = 0.15, *ϕ* = 0.03.

We now expand our investigation of TA performance by including all IDs rather than just innocent suspect IDs. A logistic regression revealed a main effect of encoded face type, (Wald (1) = 17.26, *p* < 0.001) and procedure (Wald (1) = 855.61, *p* < 0.001), but not tested face(s) type (Wald (1) = 0.002, *p* = 0.97). The 3-way interaction among these three variables was significant (Wald (1) = 11.83, *p* = 0.001), as was a 2-way interaction between procedure and tested face(s) type (Wald (1) = 13.18, *p* < 0.001). There was no interaction between encoded versus test face types (Wald (1) = 2.32, *p* = 0.13) or between encoded face type and procedure (Wald (1) = 2.13, *p* = 0.14).

Based on the significant interactions, we conducted several Chi-square analyses to investigate simple effects, beginning with showups versus lineups across the four combinations of encoded and test face type (Bonferroni-corrected alpha = 0.0125). Unsurprisingly, based on the presence of fillers, there were more IDs from lineups compared to showups across all four combinations: (a) Full-Full, *χ*^2^ (1, *N* = 2405) = 111.87, *p* < 0.001, *ϕ* = 0.22; (b) Full-Internal, *χ*^2^ (1, *N* = 2422) = 305.95, *p* < 0.001, *ϕ* = 0.36; (c) Internal-Full, *χ*^2^ (1, *N* = 2436) = 253.79, *p* < 0.001, *ϕ* = 0.32; and (d) Internal-Internal, *χ*^2^ (1, *N* = 2410) = 252.41, *p* < 0.001, *ϕ* = 0.32. Next, we compared encoded face types across the four combinations of tested face(s) type and procedure (Bonferroni-corrected alpha = 0.0125). There were more IDs after encoding an internal face compared to a full face when being tested with a full lineup, *χ*^2^ (1, *N* = 2419) = 34.18, *p* < 0.001, *ϕ* = 0.12. There was no difference between encoding a full versus internal face for any of the other combinations: (a) full showup, *χ*^2^ (1, *N* = 2422) = 0.14, *p* = 0.74, *ϕ* = 0.01; (b) internal showup, *χ*^2^ (1, *N* = 2418) = 4.28, *p* = 0.04, *ϕ* = 0.04; and (c) internal lineup, *χ*^2^ (1, *N* = 2414) = 0.12, *p* = 0.74, *ϕ* = 0.01. Last, we compared test face types across the four combinations of encoded face type and procedure (Bonferroni-corrected alpha = 0.0125). There were more IDs when tested on internal faces compared to full faces, but only after encoding a full face and tested with a lineup, *χ*^2^ (1, *N* = 2410) = 22.89, *p* < 0.001, *ϕ* = 0.10. There was no difference between internal and full face(s) at test for any of the other combinations: (a) encoding a full face and tested with a showup, *χ*^2^ (1, *N* = 2417) = 6.10, *p* = 0.014, *ϕ* = 0.05; (b) encoding an internal face and tested with a showup, *χ*^2^ (1, *N* = 2423) = 0.59, *p* = 0.47, *ϕ* = 0.02; and (c) encoding an internal face and tested with a lineup, *χ*^2^ (1, *N* = 2423) = 0.52, *p* = 0.49, *ϕ* = 0.02.

## Appendix 2: The Confidence-Accuracy Relationship

Besides discriminability, another way to discuss the concept of accuracy involves measuring its relationship with eyewitness confidence. When eyewitnesses express a higher level of confidence, does this correspond to a similar level of accuracy? Assessing the confidence-accuracy (CA) relationship in this way can permit researchers to better determine the reliability of an ID decision. Consideration of the CA relationship is certainly not new (e.g., Sporer et al. 1995), but techniques have improved to evaluate its utility (Juslin et al. 1996; Mickes 2015). The result is a growing amount of evidence suggesting that immediate confidence is typically a strong postdictor of accuracy (Wixted et al. 2015; Wixted and Wells 2017). Of course, this is not to say that it always holds for individual eyewitnesses (Sauer et al. 2019) or when best practices are not followed such as when the suspect stands out in a biased lineup (Key et al. 2017) or when a showup is used (Neuschatz et al. 2016). Even at the higher end of the confidence scale, showups have been found to still yield inferior reliability than lineups (Colloff and Wixted 2019; Mickes 2015; Wooten et al. 2020). We expected the same from our study, such that high-confidence showup IDs will not be as accurate as high-confidence lineup IDs. We also expected that the CA relationship should be somewhat impaired when there is a violation of encoding specificity. In other words, the CA relationship could be harmed by a mismatch between encoding and retrieval (e.g., Internal-Full condition). To examine the CA relationship, we used CAC (confidence-accuracy characteristic) analysis by plotting proportion correct across four bins of confidence from low (0–50%) to medium (51–70%) to high (71–90%) to very high (91–100%). Proportion correct for showups is calculated as correct IDs/(correct IDs + false IDs) and proportion correct for lineups is calculated as correct IDs/(correct IDs + [all IDs from TA lineups/6]).

See Figs. 10, 11, 12, 13, and 14.

## Footnotes

1 Differential Filler Siphoning provides another interpretation of eyewitness ID decisions (e.g., Smith et al. 2017; Smith et al. 2018; Wells et al. [Bibr CR58][Bibr CR59]), but it does not include a mechanism that would explain differences in discriminability. Rather, it is a theory about response bias, predicting more conservative suspect choosing (i.e., more filler IDs compared to suspect IDs) for lineups compared to showups (see Colloff et al. 2018), yielding a higher diagnosticity ratio for lineups (we touch on the diagnosticity ratio below). We focus our investigation on the quantitatively specified predictions from DFT, which involve discriminability.

2 DFT does not require that *all* non-diagnostic information is discounted, just that eyewitnesses give greater weight to diagnostic over non-diagnostic information. Adding non-diagnostic information merely adds noise, which could reduce discriminability somewhat (but not as much as it would for the showup in which the information is not discounted at all). Regardless of these details, in Table 2 we present a simplified version of DFT that assumes that people will be perfectly optimal in their discounting of non-diagnostic information. This assumption fits better with our manipulation of adding or subtracting large portions of the face, and also is more straightforward to instantiate in Table 2. Our predictions are largely unaffected if people are assumed to instead be less than optimal, which of course is more realistic.

3 One subtle difference between Tables 1 and 2 is the variance estimate of 1.5 for previously seen features in the original table from Wixted and Mickes (2014), which is now consistently equal to 1 for both old and new faces in Table 2. In other words, Wixted and Mickes applied the unequal-variance signal detection (UVSDT) model, whereas we switched to the equal variance model (EVSDT). The DFT predictions are the same regardless of which underlying model is specified, but we decided to apply equal variances due to the lack of support for the distribution of studied faces actually having more variance (due to encoding variability) compared to new faces (Colloff et al. 2016; Wixted et al. 2018), in contrast to the word list learning literature (e.g., Egan 1958; Mickes et al. 2007; Wixted 2007). With EVSDT, *d'* can be used in the far-right column rather than *d*_*a*_*,* but we decided to continue with the latter for continuity with Table 1.

4 As we note Table 2, for the sake of simplicity we are assuming that eyewitnesses are perfectly optimal, such that any shared non-diagnostic information (e.g., short dark hair) will be discounted, and this is only possible in a lineup and not a showup (because there is no sharing possible in a showup). We are also assuming that shared features will be discounted even if not exactly identical, such as all lineup members having short dark hair, but not the exact same hair. It turns out that our predictions do not change when these assumptions are removed, as we explain in detail in the Discussion.

5 We found no difference based on suspect position, and so all analyses below collapse over position.

6 We utilized the confidence data for two sets of analyses: (a) ROC analyses reported in the main text, as these test our hypotheses, and (b) confidence-accuracy characteristic (CAC) analysis (Mickes 2015) reported in Appendix 2, as we had no novel predictions regarding this relationship. However, we still present several CAC curves in the appendix to see whether we replicated common patterns (e.g., better relationship for lineups compared to showups) and also because the CA relationship has recently come to the forefront of the eyewitness literature (e.g., Wixted and Wells 2017).

7 There are some critics when it comes to the application of ROC analysis (and pAUC in particular; see Smith et al. [Bibr CR47][Bibr CR50]) to lineup data, who cite its collapsing of identification data into either positive (suspect IDs) or negative (filler IDs and rejections) responses as a weakness (Lampinen 2016; Wells et al. 2015a). However, in practice filler IDs are known innocents and are therefore less consequential than suspect IDs. We are confident in our reliance on ROC analysis based on pAUCs, as this has become the primary method of determining differences in discriminability between ID procedures in the literature. Moreover, the arguments against applying ROC analysis (of pAUCs) to lineup data (e.g., Lampinen 2016; Wells et al. 2015a; Wells et al. [Bibr CR58][Bibr CR59]) have been thoroughly addressed by others (e.g., Rotello and Chen 2016; Wixted and Mickes 2015a,[Bibr CR66][Bibr CR67]; Wixted et al. 2017) and are beyond the scope of our paper.
